# Metacognitive Confidence Increases with, but Does Not Determine, Visual Perceptual Learning

**DOI:** 10.1371/journal.pone.0151218

**Published:** 2016-03-16

**Authors:** Leopold Zizlsperger, Florian Kümmel, Thomas Haarmeier

**Affiliations:** 1 Division of Vascular Neurology and Neurorehabilitation, Department of Neurology, University Hospital and University of Zürich, Zürich, Switzerland; 2 cereneo Center for Neurology and Rehabilitation, Vitznau, Switzerland; 3 Cognitive Neurology, Hertie Institute for Clinical Brain Research, Tübingen, Germany; 4 Department of Neurology, RWTH Aachen University, Aachen, Germany; 5 Department of Neurology, HELIOS Clinic Krefeld, Krefeld, Germany; University of Melbourne, AUSTRALIA

## Abstract

While perceptual learning increases objective sensitivity, the effects on the constant interaction of the process of perception and its metacognitive evaluation have been rarely investigated. Visual perception has been described as a process of probabilistic inference featuring metacognitive evaluations of choice certainty. For visual motion perception in healthy, naive human subjects here we show that perceptual sensitivity and confidence in it increased with training. The metacognitive sensitivity–estimated from certainty ratings by a bias-free signal detection theoretic approach–in contrast, did not. Concomitant 3Hz transcranial alternating current stimulation (tACS) was applied in compliance with previous findings on effective high-low cross-frequency coupling subserving signal detection. While perceptual accuracy and confidence in it improved with training, there were no statistically significant tACS effects. Neither metacognitive sensitivity in distinguishing between their own correct and incorrect stimulus classifications, nor decision confidence itself determined the subjects’ visual perceptual learning. Improvements of objective performance and the metacognitive confidence in it were rather determined by the perceptual sensitivity at the outset of the experiment. Post-decision certainty in visual perceptual learning was neither independent of objective performance, nor requisite for changes in sensitivity, but rather covaried with objective performance. The exact functional role of metacognitive confidence in human visual perception has yet to be determined.

## Introduction

Visual perceptual learning enhances perceptual sensitivity, enabling us to adapt to new sensory environments [1,2]. While subjective awareness of the content of perception also increases with training [3], metacognitive reports about the *process* of cognition [4]–e.g. the confidence in a perceptual decision–surprisingly have been rarely investigated systematically. Since sensory information is noisy and insufficient to uniquely determine the environment, natural perceptual systems evolved to cope with systematic uncertainty. Accordingly, visual perception has been described as a process of inference by which ambiguous sensory cues are combined with internal knowledge of the world to arrive at an interpretation of the scene that is most likely to be true [5,6]. Theoretical and experimental data suggest that the underlying neural computations approximate a form of probabilistic reasoning featuring choice certainty in a Bayesian statistical framework [7,8]. Decision confidence in this context has been specified as a probabilistic estimate of past performance and expected outcome [8,9,10]. But how does subjective confidence change in the course of visual perceptual learning? Perceptual confidence is considered to be among the characteristics of a decision process that influence successful choice formation and enactment [11]. Subjective certainties of both previous knowledge and current sensory input determine their optimal weighting in every perceptual decision [12]. The exact role of subjective confidence in perceptual learning, however, has yet to be determined. Presumably, improvements in sensitivity and decision confidence depend on each other. Are changes in perceptual sensitivity a prerequisite for changes in subjective confidence in the course of visual learning? Or does decision confidence rather serve as a”ruler”that guides perceptual learning? A certain degree of subjective confidence in a perception could be necessary for changes in sensitivity to occur. Previous findings suggested that low-level inputs [13] and high-level cognitive processes like selective attention [14] or performance feedback [15] gate perceptual learning. Visual perceptual learning has been shown to be most effective when the target was successfully recognized suggesting that successful recognition triggers a learning signal [16], perhaps a sense of accomplishment that serves as an internal reward.

We hypothesize that metacognitive evaluations of one’s performance quasi calibrate subsequent perceptual learning. To differentiate subjective perceptual confidence estimates and actual metacognitive sensitivity [17,18], we here applied a non-parametric approach based on signal detection theoretic receiver operating characteristic statistics. This procedure has been shown to reliably separate metacognitive sensitivity from undesirable influences such as individual response bias [18]. While confidence and bias fluctuate from trial to trial, the observers’ metacognitive sensitivity is calculated with reference to the external world by the experimenter and is supposed to be rather constant for the experimental combination of individual subject and stimulus. In our present learning experiment we thus examined if metacognitive sensitivity–how good the individuals are at distinguishing between their own correct and incorrect stimulus classifications–serves as a ruler for subsequent visual perceptual performance.

In a different line of research there has been a gathering consensus on the causal role of brain oscillations in a variety of cognitive functions [19,20], particularly learning [21]. Recent data have suggested an oscillatory hierarchy with faster oscillations being locked to preferred phases of underlying slower waves in cognitive processing [22,23]. Multiunit activity recordings in the alert monkey visual cortex [24] and human magnetoencephalography (MEG) [25] established phase-locking of occipital high-frequency oscillations in the gamma range (63±5 Hz) to a slow-frequency oscillation in the delta band (1–5 Hz), with the strength of gamma amplitude modulation reflecting the success in visual discrimination [25]. This correlation provided further evidence for the hypothesis that coupling between low- and high-frequency brain oscillations subserves signal detection. These findings encouraged us to include a targeted 3 Hz transcranial alternating current stimulation (tACS) in the present experiment aiming to enhance visual perceptual learning. As there is compelling evidence that phase dynamics reflect cyclic fluctuations of neural excitability and play a relevant functional role in cognitive processes [26,27], the stimulation parameters were alternated to mimic endogenous delta oscillations with a phase angle relative to stimulus onset of 0°, 180°, or sham stimulation, respectively.

In previous studies we demonstrated that the confidence in a perceptual decision dissociates from perceptual sensitivity with selective attention [9] and that electroencephalographic correlates of decision confidence can be disentangled from representations of sensory evidence, objective discrimination performance and overt motor behavior [10]. For a variation of this established visual motion perception paradigm here we explored in healthy, naive human subjects how objective performance and its metacognitive evaluation develop and interact in the time course of perceptual learning. Complemented by the opportunity of exogenously modulating oscillatory cortical network activity − based on *a priori* MEG knowledge [25]–we aspired to further illuminate the role of decision confidence in perceptual learning. Since pathological changes of metacognition are pervasive in many neuropsychological disorders [28,29], a deeper understanding of its function in perceptual learning promises new strategies and tACS protocols to improve learning and optimize relearning and rehabilitation.

## Materials and Methods

### Subjects

30 healthy subjects (20 females) with a mean age of 31 years were tested. The sample size was chosen to exceed the number of subjects in previous transcranial stimulation [30,31] and psychophysical studies on human perceptual learning of visual motion discrimination [32,33,34] in order to detect significant effects in both domains. 29 participants were right-handed, one was left-handed, all had normal or corrected-to-normal visual acuity. Subjects reported to be free of neurological or psychiatric impairment and showed no risk factors for tACS application as assessed through safety questionnaires (such as neurological, psychiatric or cardiological disorders, intracranial metal). Written informed consent was obtained from all subjects according to the Declaration of Helsinki and the guidelines of the local ethics committee of the faculty of medicine of the University of Tübingen, which approved the procedures.

### Design and procedures

**Fig 1:**
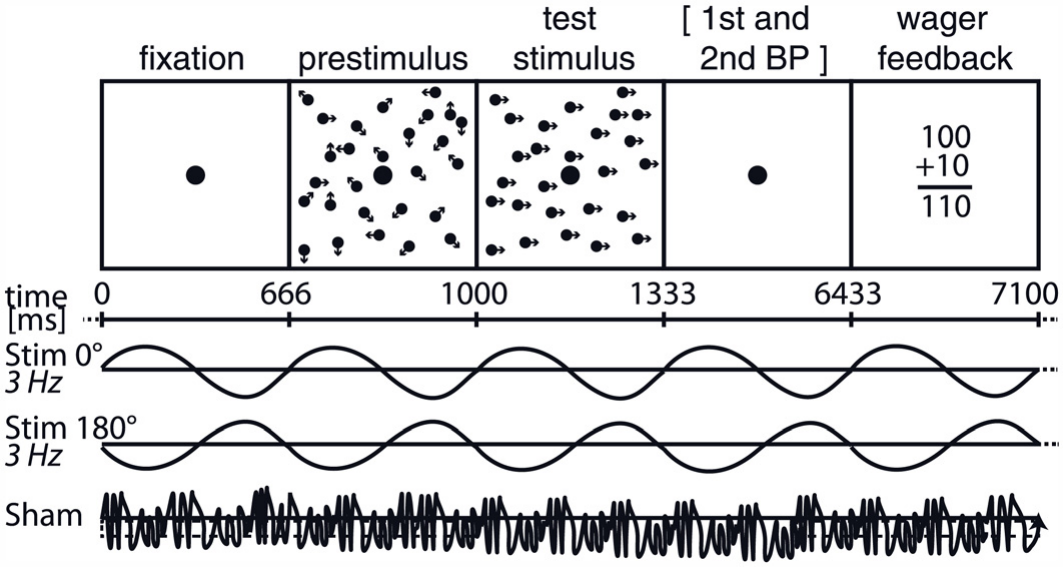
Timeline of the behavioral task and transcranial alternating current stimulation (tACS). The test stimulus consisted of a random dot kinematogram presented at the center of the screen while eye movements were monitored (1000–1333 ms). The level of motion coherence and the direction of global motion (four alternatives) were modulated on a trial-by-trial basis. Subjects reported perceived motion direction with a first button press and decision certainty with a second button press corresponding to a high (10) or low (1) wager. Wager feedback was given via a continuously updated point score adding or subtracting the chosen virtual bet. As symbolized by the three different stimulation tracks below the stimulus schematic, tACS was delivered in three sessions using either 3 Hz stimulation with a phase angle of 0° or 180° relative to stimulus onset, or sham stimulation. The sequence of the sessions was permutated, the subjects were equally randomized to the resulting six groups.

To explore objective and subjective performance, a variation of a random dot stimulus we used before [25,35] was presented. Subjects were instructed to discriminate the global direction of a motion stimulus surrounding a central fixation dot while eye movements were monitored, 30-channel EEG was recorded and tACS was applied as per the stimulation protocol detailed below. Both a decision and the confidence in it was given trial by trial. The visual stimulus consisted of five periods (Fig 1): A first fixation period (central fixation dot, black, diameter 2 arcmin) that lasted 666 ms was followed by a random dot kinematogram (RDK) covering a square of 9° x 9° centered around the fixation point. This prestimulus consisted of 475 white squares (side length = 0.8 arcmin, lifetime = 500 ms, dot density ~6 dots/deg^2^, luminance 384 cd/m^2^) on a black background (luminance 0.14 cd/m^2^). All dots were moving incoherently, that is, in all possible directions with a resolution of 1°, at a common speed of 6 deg/s. After 333 ms a second RDK, the test stimulus, started [35,36]. The properties of the test stimulus were identical to those described for the prestimulus except that a certain percentage of the dot elements moved coherently in the same direction (either up, right, down or left). The prestimulus RDK was introduced to separate the EEG response reflecting visual motion onset and the inherent luminance changes from the electrophysiological activity following the test stimulus. We successfully used this four-alternative forced-choice task in previous studies on perceptual decision confidence [9,10] as it has been demonstrated to be the preferred method of determining psychophysical thresholds in naive observers [37]. In one half of the trials the percentage of coherently moving dots in an individual trial was chosen equally and randomly from four predefined steps (0%, 15%, 35%, or 100% of all dots), in the other half the coherence level was chosen according to an adaptive staircase procedure [38,39]. Start level of this procedure was 80% coherence. The change in coherence started with a step of 20% and step size was halved in case the last change had caused convergence toward the 62.5% correct threshold. In our four-alternative forced choice task this threshold corresponds to subjects being correct in half of the trials. Step size was doubled (if possible) in case the change had led further away from this threshold. The staircase procedure was terminated and started new when the step size had reached a value of 0.02%. A combined approach was chosen to improve both the fit of the probit function to the individual psychometric data (staircase procedure) and the comparability of datasets between subjects (four predefined coherence levels). Following stimulus offset, subjects had to report the direction of coherent motion. Post-decisional certainty was indicated by means of post-decision wagering [40], an intuitive way to measure metacognitive certainty as subjects place virtual bets on their previous performance [40]. Subjects were instructed to place a wager of either 1 or 10 virtual points by pressing the left (1) or right (10) button on the interface device after their direction choice. They would win or lose this amount depending on whether their first choice was correct or incorrect. Subjects started with an amount of 100 virtual points. In our instructions to the participants we avoided to give the wager a financial connotation or association to any kind of reward in order to minimize the confounding effects of economic variables reported in previous studies [41,42]. Subjects were not given a compensation after completing the experiment. Feedback was given at the end of the fixed 5100 ms response window by showing a central counter (dimensions: 7.5°x6°, white, luminance 384 cd/m^2^) for 666 ms. Due to the trial-by-trial wager feedback perceptual learning in this experiment best fits a task-irrelevant reinforcement learning model [2]. Subjects viewed all stimuli binocularly from a distance of 55 cm on a 19” TFT-display (native resolution 1280x1024 pixels) driven by a Linux computer running the nrec visual stimulation, data acquisition and experiment control software package (http://nrec.neurologie.uni-tuebingen.de, created by F. Bunjes, J. Gukelberger et. al.) at a refresh rate of 60 Hz in a dark, quiet room.

### Eye movement recordings

During the entire course of a single trial subjects would fixate the central fixation dot while eye movements were monitored using a custom built video system taking the pupil’s center as measure of eye position. Recordings were stored at a sampling rate of 50 Hz and quality of fixation was analyzed offline. In particular, deviations from the fixation point (eye position) were examined for the period of test stimulus presentation. All subjects maintained stable fixation as indicated by a mean horizontal (h) and vertical (v) eye positions close to 0° and small standard deviations: h: 0.87° ± 0.04°, v: 0.50° ± 0.04°.

### Transcranial alternating current stimulation–tACS

tACS was delivered by a battery-driven current stimulator (neuroConn GmbH, Ilmenau, Germany) through conductive-rubber electrodes (5 cm x 7 cm = 35 cm^2^) placed in saline-soaked sponges. While EEG was recorded at 29 scalp sites, for details see below, the target electrode was placed centrally over the occipital area (between electrodes O1 and O2) and the reference electrode was positioned centrally over the frontal area (between electrodes Fp1 and Fp2). The electrodes Fp1, Fp2, O1, and O2 each had been moved laterally by about two centimeters to accomodate the stimulation electrodes underneath the EEG cap without establishing contact with the sponges. Target electrode position and stimulation frequency were selected with the results of a previous study in mind that demonstrated MEG cross-frequency coupling of delta and gamma oscillations over occipital sensors suggesting a functional role of oscillatory fluctuations in the delta band in signal detection [25]. A sinusoidal electrical current waveform was applied at a frequency of 3 Hz. An intensity of 1.5 mA was chosen to avoid the perception of flickering lights usually reported with higher stimulation intensities [43]. With evidence for periodic sampling of visual perception as a function of the phase of ongoing EEG oscillations [44], the phase angle of the 3 Hz stimulation relative to stimulus onset was varied for the two stimulation blocks. In a first condition 3 Hz stimulation started with a phase angle of 0°, in a second with 180°. In a third block subjects received sham stimulation with a normally distributed broadband low and high frequency noise signal of 1.5 mA. In all three conditions tACS was applied during the entire course of each stimulation trial. All participants completed the training session and the three experimental blocks during the same day, the sequence of the blocks was permutated resulting in six groups the subjects were equally randomized to. All subjects were naive to tACS effects and did not know which stimulation group they were assigned to. There were 220 trials in each block, all subjects completed all three blocks with five-minute breaks between blocks. At the beginning subjects performed a short standardized training session to familiarize themselves with the task (10 trials). During stimulation EEG was recorded at 29 scalp sites as determined by the International 10–20 EEG system (Fp1, Fp2, F3, F4, C3, C4, P3, P4, O1, O2, F7, F8, T7, T8, P7, P8, Fz, Cz, Pz, FC1, FC2, CP1, CP2, FC5, FC6, CP5, CP6, TP9 and TP10) versus the additional FCz electrode, using an electrode cap equipped with Ag/AgCl electrodes (Easycap, Herrsching, Germany). The electrooculogram (EOG) was monitored using an additional electrode placed below the lateral canthus of the right eye. The impedance of the electrodes was kept below 10 kΩ, EEG and EOG signals were amplified using a 32-channel BrainAmp EEG amplifier (Brainproducts, Munich, Germany). With regard to the simultaneous EEG recording the stimulation or sham signal was applied only in every second trial, aiming to leave half of the trials without EEG artifacts due to tACS. Unfortunately, due to the relatively short trial duration of 5100 ms stimulation artifacts exceeded the effective stimulation interval making the interspersed trials ineligible for further electrophysiological analysis.

### Data analysis

The psychometric functions of choice accuracy and certainty were plotted for all trials (fixed coherence and staircase) as proportion of correct decisions and high wagers, respectively, against the coherence level of the motion signal. To compare accuracy and certainty data psychophysically, a certainty index was defined that reflects the stochastics of the task: Low wagers were assigned a value of 0.25 matching chance level, high wagers a value of 1 matching perfect discrimination. Accuracy and certainty each were fitted using a probit function [9,45]. The objective and subjective perceptual thresholds were defined by the coherence level for which the respective probit function predicted 62.5% correct responses. Thresholds were determined for three consecutive sections of 73 trials each within a stimulation block–resulting in nine consecutive learning sections–and for the stimulation block as a whole. The last trial in each stimulation block was discarded. To verify whether and how accuracy and certainty were affected by tACS, objective and subjective thresholds were submitted to a two-way ANOVA with the within-subject factors type of stimulation (3 Hz tACS phase 0°, 3 Hz tACS phase 180°, sham) and order of the three blocks. Correlation, linear and partial regression analyses were performed to investigate the evolution of accuracy, certainty, and their interaction. Where the examination of incremental learning effects by means of linear regression or correlation analyses required a control for repeated measures we implemented a two step approach proposed for this purpose previously [46]. In the first phase of this procedure, separate regression equations were computed for each subject in the experiment. In the second phase of the analysis we tested via single-group *t*-tests if the regression coefficients differed significantly from zero. The results of this control are specified along with the results of the single regression analyses on the entire data sets.

To quantify how precise subjects assessed their objective performance we examined their metacognitive sensitivity via a non-parametric signal detection theoretic approach (SDT) that is widely used in the literature on perceptual metacognition (for a recent review see [47]). Based on each subject’s confidence estimates we performed a receiver operating characteristic (ROC) analysis following the authors’ suggestions in [18] and the provided Matlab code. In SDT analyses the subjects’ performance is determined by comparing the proportion of “hits” and “false alarms” in a stimulus detection task. Transferring this logic of SDT to metacognition (type 2 SDT), a “hit” was categorised as a high confidence response after a correct decision and a “false alarm” as a high confidence response after an incorrect decision. A higher area under the type 2 ROC curve (AUROC2) indicated higher metacognitive sensitivity. This non-parametric method provides a bias-free measure of the connection between perceptual performance and metacognition by quantifying how well wagers differentiate between correct and incorrect trials. On a trial-by-trial basis this approach assesses the relationship between wagering and performance irrespective of the subject’s proclivity toward high or low wagers.

AUROC2 was calculated for each subject separately based on the certainty estimates of all presented trials with a motion coherence level other than 0%.

To obtain data using a constant level of task difficulty or stimulus strength subjects were presented with the identical motion coherence levels in predefined steps and number of trials in the task, supplemented by data on individual near-threshold performance due to the adaptive staircase procedure.

In a next step we investigated what exactly determined successful perceptual learning. We quantified the learning effect by the difference of the initial accuracy threshold in section one of nine and the respective threshold in the last of the nine sections. This learning effect was correlated to initial accuracy, certainty, and metacognitive sensitivity measures of section one of nine. As the naive subjects were new to the experimental paradigm and completed only ten trials to get familiar with the task, we added a control for the possible confounder of early procedural learning. In order to rule out that at the beginning participants were mainly mastering the requirements of the task, explaining the bulk of the behavioral improvement, performance in section one was disregarded and the correlation analysis was repeated for overall learning from section two to nine. Since accuracy and certainty thresholds correlated significantly in the course of the experiment, partial correlation analyses were added where necessary to control for the covariance of accuracy and certainty. In a final step we examined the learning effect in a more fine-grained analysis: We observed the impact of each of the first eight section’s accuracy, certainty, and metacognitive sensitivity measures on the improvement of objective performance that immediately followed each section. This gradual evolution with practice was quantified by the eight accuracy threshold deltas of section n minus section n+1. This incremental effect, again, was analyzed via correlation and partial correlation analyses.

## Results

In the present experiment we examined how objective performance, subjective confidence in it, and metacognitive sensitivity change in the course of visual perceptual learning. 30 subjects that were healthy and naive to the task completed a random dot motion discrimination task while trial-by-trial performance feedback was given and 3 Hz tACS or sham stimulation was applied concomitantly. Participants did not feel any relevant discomfort during occipital tACS as per their spontaneous reports at the end of the experiment. As expected for delta frequency stimulation and a current of 1.5 mA no retinal phosphenes were reported [43,48].

**Fig 2:**
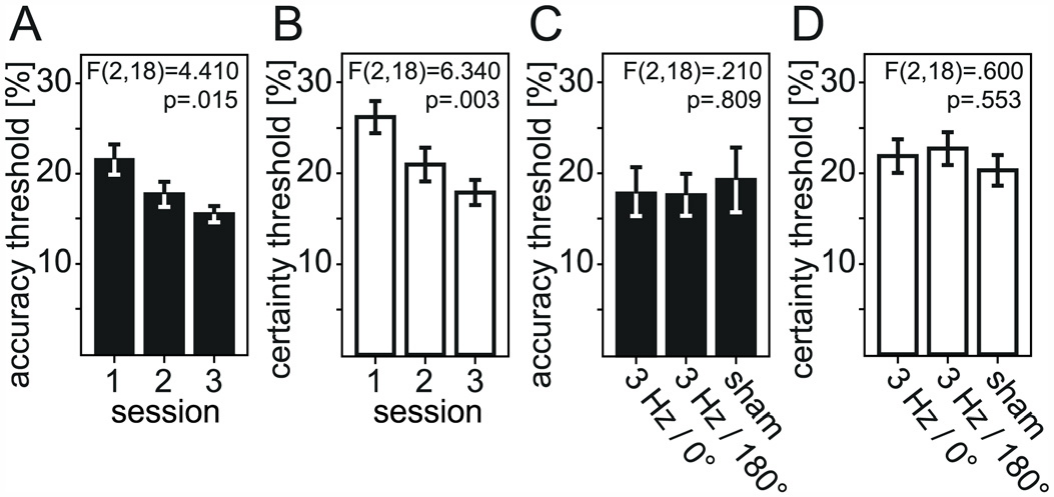
Perceptual accuracy and choice certainty, tACS sessions. (A) Proportion of correct responses and (B) certainty index for the three experimental sessions, chronological order. (C) Proportion of correct responses and (D) certainty index for the three experimental sessions by type of tACS stimulation. Data are mean of all subjects ± s.e.m.

**Fig 3:**
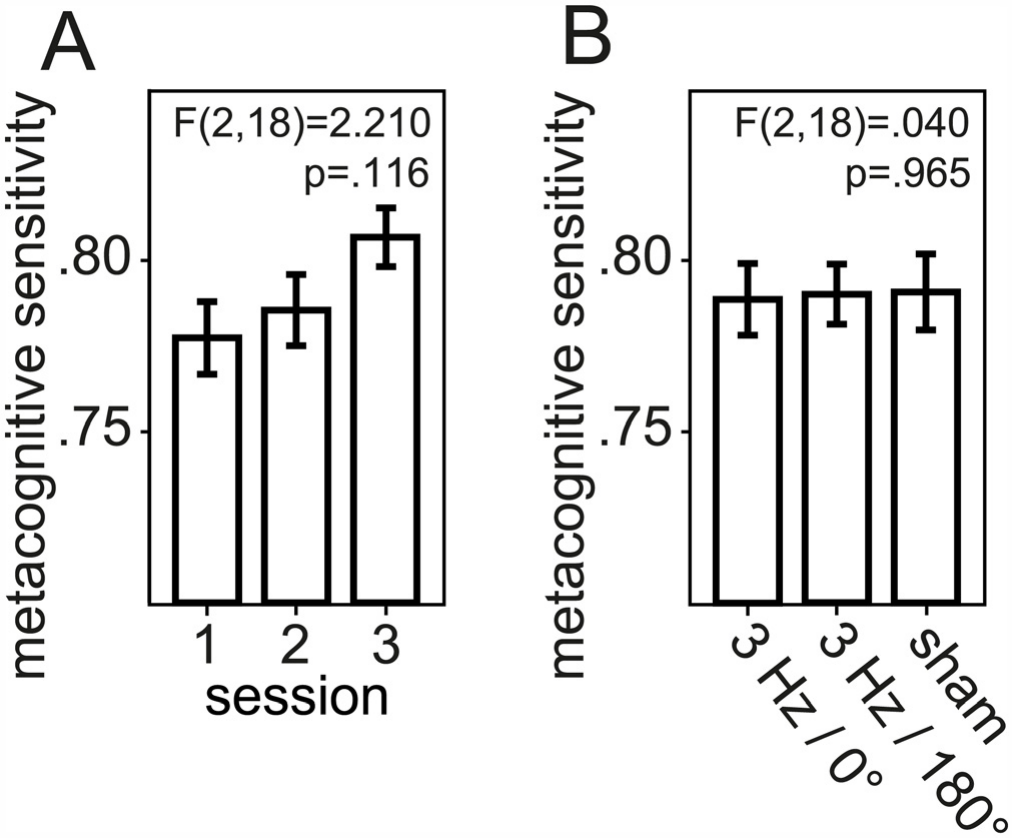
Metacognitive sensitivity, tACS sessions. Mean metacognitive sensitivity for the three experimental sessions: (A) chronological order, (B) by type of tACS stimulation. Metacognitive sensitivity was determined by a non-parametric signal detection theoretic approach, in which a higher area under the type 2 ROC curve (AUROC2) indicated higher metacognitive sensitivity. Data are mean of all subjects ± s.e.m.

Discrimination performance and perceptual decision confidence increased in the course of the experiment as indicated by decreasing accuracy and certainty thresholds (Fig 2A and 2B). The motion sensitivity threshold indicated the coherence level for which a probit function fitted to the data predicted 62.5% correct responses. The confidence threshold specified the corresponding value for a fit to the certainty index that reflect the stochastics of the task and subjects’ instructions, cf. *Material and Methods*. The mean accuracy threshold of 21.56% in the first of the three sessions went down to 15.49% in the last session resulting in a 28% improvement referred to the initial performance. Analogously, certainty thresholds improved by 32% compared to the initial confidence (from a mean of 26.21% to 17.88%). When tested for the influence of the type of stimulation (3 Hz tACS with 0°, 180° phase, or sham) and the timing in the course of the experiment (first, second, or third session) by a 2-way repeated measures ANOVA, both discrimination accuracy (F(2,18) = 4.410; p = .015) and the subjective confidence in the perceptual decision (F(2,18) = 6.340; p = .003) increased significantly with training. As illustrated in Fig 2C, discrimination accuracy thresholds averaged out at 17.93% for 0° phase, 17.57% for 180° phase, and 19.24% for sham stimulation. Averaged choice certainty thresholds were at 21.99% for 0° phase, 22.74% for 180° phase, and 20.32% for sham stimulation (Fig 2D). As expected from these threshold values, accuracy and certainty did not differ significantly for the different modes of transcranial current stimulation (F(2,18) = .210; p = .809 for accuracy and F(2,18) = .600; p = .553 for certainty). Since the mean certainty thresholds theoretically might be influenced by the individual subject’s propensity to indicate on average higher or lower confidence, we next sought to analyze a bias-free measure of how good the individuals actually distinguished between their own correct and incorrect stimulus classifications. This measure of metacognitive sensitivity–type 2 sensitivity based on a signal detection theoretic appproach detailed in *Material and Methods*–was examined for the influences of tACS and the time course of learning via a 2-way repeated measures ANOVA, as well. The area under the type 2 receiver operated characteristics curve (AUROC2) averaged out at .78 for 0° phase, .79 for 180° phase, and .81 for sham stimulation (Fig 3B). The mean AUROC2 was .78 in the first, .79 in the second, and .81 in the third of the three learning sessions (Fig 3A). Metacognitive sensitivity as quantified by these AUROC2 measures did not differ significantly for the different modes of transcranial current stimulation (F(2,18) = .040; p = .965) or the timing in the course of the experiment (F(2,18) = 2.210; p = .116). Responses were given significantly faster in the course of the experiment (F(2,16) = 5.726; p = .013), while the type of stimulation did not influence RTs significantly (F(2,16) = .948; p = .408). The influence of tACS consequently was not considered in the further analyses of this learning experiment.

**Fig 4:**
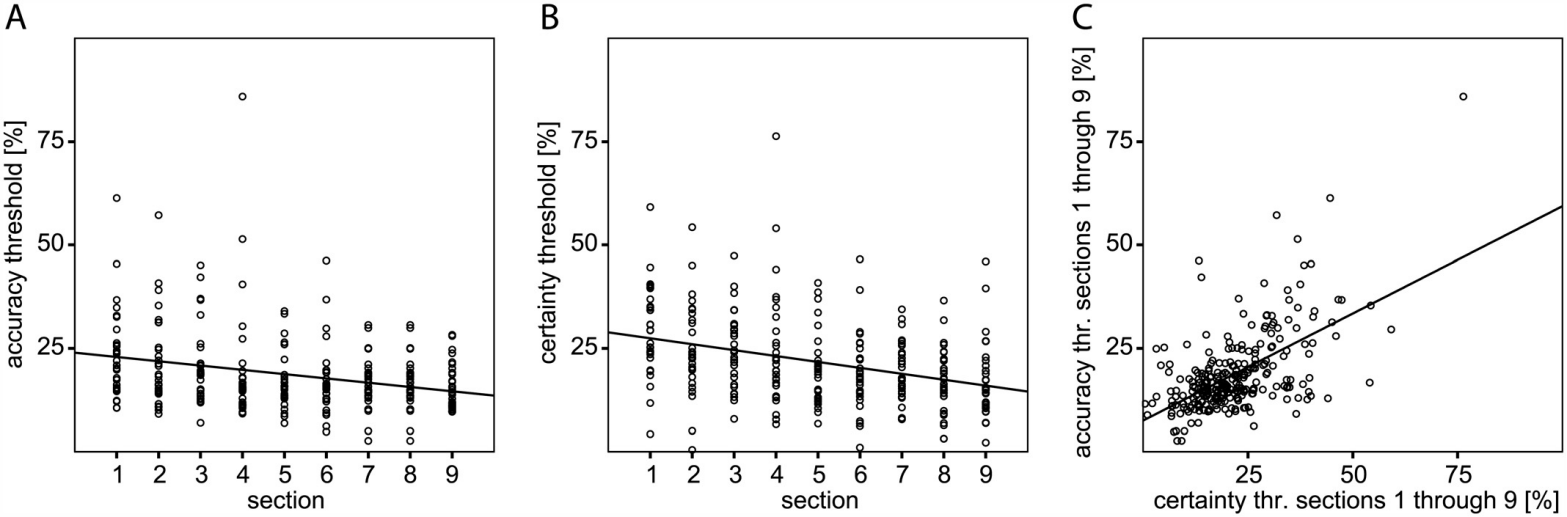
Perceptual accuracy, choice certainty, and their correlation through the nine consecutive learning sections. (A) Each subject’s proportion of correct responses and (B) certainty index for the nine consecutive experimental sections. (C) Correlation of these accuracy and certainty thresholds through the time course of the experiment (nine sections), all subjects.

To sample the development of objective and subjective performance with perceptual learning at closer intervals, each of the subjects’ three experimental stimulation sessions was divided into three sections subsequently. This resulted in nine consecutive learning sections of 73 trials we calculated accuracy and certainty thresholds for. In the course of the experiment all subjects–who started without previous training–improved their objective performance as indicated by decreasing perceptual thresholds (Fig 4A). In other words, subjects progressively managed to correctly indicate global motion direction for lower coherence levels. Decision confidence also increased with perceptual learning (Fig 4B). Linear regressions revealed that perceptual accuracy (Beta = -.302; p < .001; controlling for repeated measures (RMC): p < .001) and certainty thresholds (Beta = -.360; p < .001; RMC: p < .001) significantly decreased with practice, accordingly. Fig 4C illustrates that post-decision certainty closely correlated with discrimination accuracy in our learning experiment (R = .597; p < .001; RMC: p < .001). Metacognitive sensitivity was not analyzed in this more fine-grained analysis across the nine learning sections, as the sequence of the original three experimental sessions did not impact it significantly in the first place.

**Fig 5:**
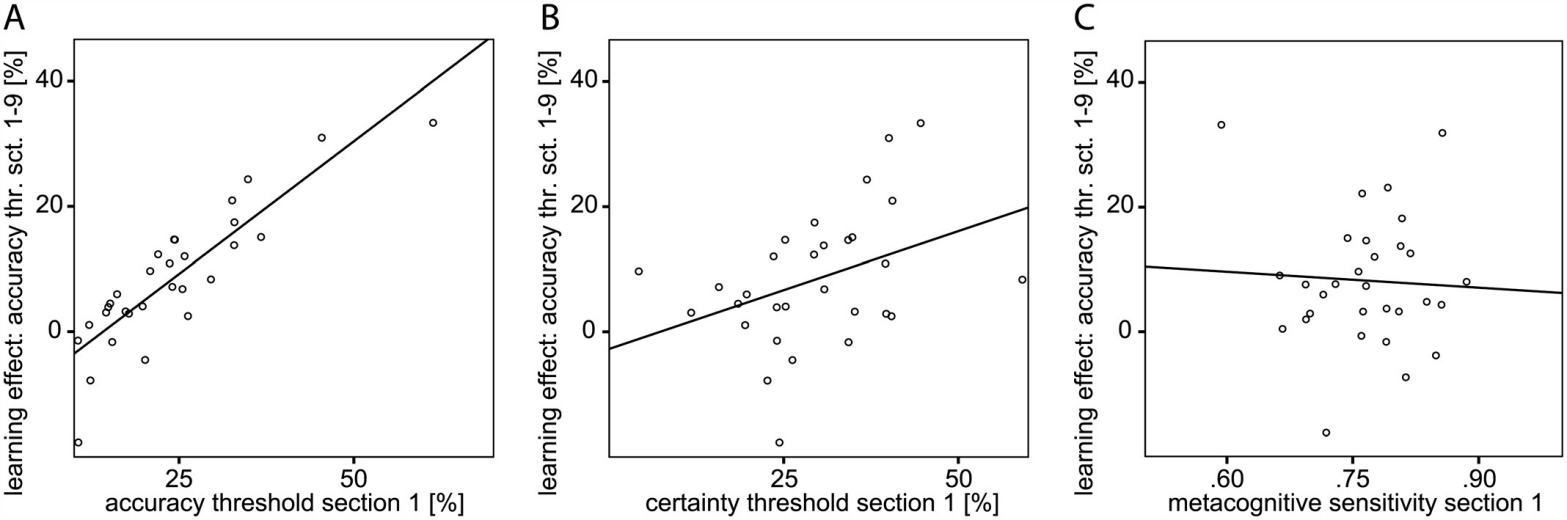
Determinants of the overall perceptual learning effect. Each subject’s overall learning effect as a function of the first (of nine) section’s (A) accuracy threshold, (B) certainty threshold, and (C) metacognitive sensitivity. The overall learning effect was parametrized as the difference between the perceptual accuracy threshold of section one and nine.

In a next step, we aimed to clarify which factors determine successful perceptual learning. The subject’s initial discrimination accuracy, its metacognitive evaluation, and metacognitive sensitivity each may well indicate to what extent perceptual performance is going to improve. Good initial motion detection, for instance, could act as an early indicator for a pronounced learning effect. On the other hand, it might as well signify that there is not much room left for further improvement. For statistical testing, accordingly, we quantified this learning effect by the difference of the initial accuracy threshold in section one of nine and the respective threshold in the last of the nine sections. Analyzed separately, linear regression showed a significant interaction of the learning effect with initial discrimination accuracy (Beta = .863; p < .001; Fig 5A) and confidence in it (Beta = .376; p = .040; Fig 5B). The correlation analysis was repeated for overall learning from section two to nine in order to rule out that participants initially were mainly mastering the requirements of the task and procedural learning was predominant at the outset. Disregarding the participants’ performance in section one completely, overall perceptual learning still was significantly determined by discrimination accuracy at the beginning (Beta = .867; p < .001). Considering that accuracy and certainty thresholds evolved quite concordantly, the question arose as to whether the two performance measures are interdependent. We verified a significant interaction of accuracy and certainty thresholds in the course of the experiment via a linear regression analysis (Beta = .604; p < .001; Fig 4C). In other words, subjects with lower accuracy thresholds–i.e. better motion discrimination–had lower certainty index thresholds–i.e. more confidence in their perceptual decision.

Does better objective performance induce higher confidence or does high subjective certainty enhance motion discrimination? A third option is a mutual interaction of both performance measures. To answer these questions we added controls for the covariance of accuracy and certainty. In a partial correlation analysis the degree of association of the learning effect and choice confidence or perceptual accuracy, respectively, was examined, with the effect of the other variable removed. Sensitivity at the outset of the experiment alone correlated significantly with perceptual learning (R = .850; p < .001), while the initial certainty did not determine the learning effect (R = -.253; p = .186). The initial metacognitive sensitivity did not determine later perceptual learning either (Beta = -.084; p = .660; Fig 5C). Successful learning hence correlated with lower sensitivity to the motion stimulus at the beginning of the experiment. The significant correlation of successful learning with the initial perceptual certainty was based on covariance with the initial perceptual accuracy.

**Fig 6:**
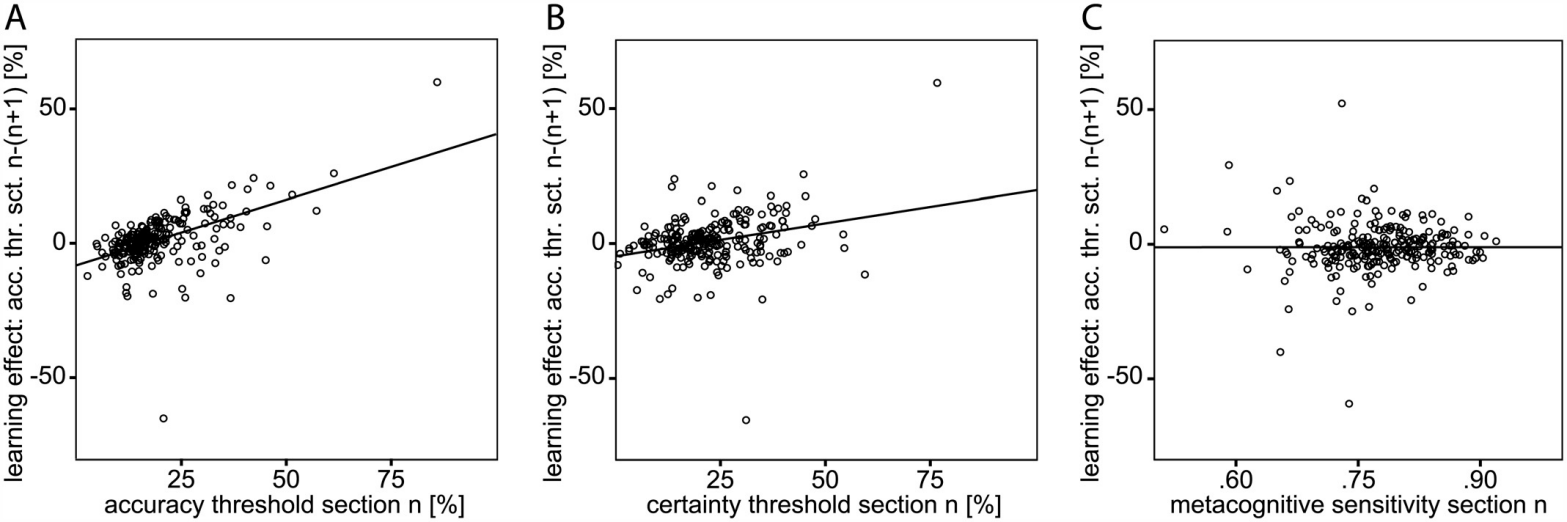
Determinants of the incremental perceptual learning effect. Each subject’s incremental learning effect as a function of the preceding section’s (A) accuracy threshold, (B) certainty threshold, and (C) metacognitive sensitivity. The incremental learning effect was parametrized as the difference between the perceptual accuracy threshold of section n and section n+1 and related to the mean accuracy threshold, certainty threshold, or metacognitive sensitivity of section n.

In a final step we conducted a more fine-grained analysis of the learning effect. We observed the impact of each of the first eight section’s accuracy, certainty, and validity measures on the improvement of objective performance that immediately followed the respective section. This gradual evolution with practice was quantified by the eight accuracy threshold deltas of section n minus section n+1. All effects observed earlier were also present when perceptual learning was analyzed at a finer time scale. Linear regression analyses verified that the preceding accuracy (Beta = .523; p < .001; RMC: p < .001; Fig 6A) and certainty thresholds (Beta = .297; p < .001; RMC: p < .001; Fig 6B) interacted significantly with the incremental learning effect, while the metacognitive sensitivity did not (Beta = .024; p = .714; RMC: p = .074; Fig 6C). Yet again, in a partial correlation analysis the incremental perceptual performance improvements with learning correlated with the motion sensitivity in the section that preceded it immediately (R = .451; p < .001; RMC: p < .001), but not with a section’s mean confidence (R = -.020; p = .758; RMC: p = .978).

## Discussion

In our combined learning and tACS experiment here we found that both objective discrimination performance and the subjective confidence in it changed in perceptual learning, but tACS did not reliably influence performance in our visual task (cf. Fig 2A and 2B vs. Fig 2C and 2D). Metacognitive sensitivity did not change significantly with tACS either (Fig 3B). Before we discuss the behavioral learning effects in detail we would like to speculate why the applied stimulation did not affect performance measures. The present study was designed to exploit our previous MEG findings establishing that the amount of cross-frequency coupling relates to the success in visual discrimination with increased strength of delta-gamma frequency coupling accentuated over the occipital pole for correctly compared to incorrectly answered trials [25]. In addition, monkey electrophysiology demonstrated delta oscillations in V1 that reflect a mechanism of internally generated cyclical variations in the excitability of a neuronal ensemble amplifying subthreshold inputs [24]. Based on that evidence we decided to apply 3 Hz tACS over the central occipital area stimulating during every second trial to achieve an increasing effect over the course of the experiment. It is possible that the stimulation intervals have been too short to build up an effect and a continuous stimulation for a longer period of time would have been more effective. Additionally, poor spatial resolution of transcranial electric stimulation when using rectangular-pad electrode configurations might also have prevented the necessary focality and modulated cortical areas adjacent to the target site [49]. We varied the tACS phase angle of the 3 Hz stimulation relative to stimulus onset for the two stimulation blocks (0° and 180°), but it may well be that tACS did not influence cross-frequency coupling adequately. Ideally, tACS application should be triggered by the temporal dynamics of the phase of the underling brain oscillations [50]. On the other hand, frequency-specific stimulation might have been effective, but was attenuated by the distinct power of occipital delta activity, since the power of the endogenous oscillations has a critical impact on tACS efficacy [51]. Due to the technical limitations of our one-on/one-off stimulation the concurrent EEG recordings were too contaminated with artifacts distorting the electrophysiological signal to verify potential delta entrainment by tACS. Without confirmation of direct entrainment of brain oscillations induced by tACS, our data cannot prove that tACS is unable to manipulate EEG oscillations. In a first conclusion, our findings did not provide decisive evidence for 3 Hz tACS reliably inducing direct modulations of visual motion discrimination and the confidence in the perceptual decision. While tACS entrainment of slow cortical oscillations (0.5–1 Hz) has been verified across frontocentral cortical areas in healthy human subjects via concurrent EEG recordings [52], a PubMed research did not return further evidence in the present literature for successful entrainment of delta oscillations in humans or for delta tACS over visual areas improving perceptual performance. Accordingly in our main analysis we focused on the evolution of psychophysical discrimination performance and the confidence in it in the course of perceptual learning.

We found that both motion discrimination sensitivity and subjective confidence in that perceptual decision increased in perceptual learning. While this improvement with training is an established finding for motion discrimination in monkeys [53] and humans [54], there has been no evidence so far on the evolution of decision confidence with learning. Previous data showed that subjective awareness–a metacognitive report about the *content* of cognition–alone is not sufficient for improving or maintaining objective performance: Dissociations of sensitivity and subjective awareness have been reported for visual perceptual learning in motion processing [55], metacontrast-masking shape-discrimination [3], or sinusoidal grating orientation [56]. Selective attention by itself does not suffice to enhance perceptual learning performance either [57]. We rather hypothesized in contrast that in perceptual learning metacognitive evaluations of the *process* of cognition, decision confidence in our experiment, interact with objective performance.

The present findings demonstrated that changes in objective performance were accompanied by changes in subjective confidence in it, suggesting that the stimulus representation was accessible for subjective report. Learning affected these two aspects of visual perception similarly with an accordant improvement of motion discrimination and perceptual choice certainty (Fig 4A and 4B): Accuracy and certainty thresholds closely correlated in the course of learning (Fig 4C). Apparently, subjects got more confident as their perceptual sensitivity improved with training. Post-decision certainty in perceptual learning was not independent of objective performance. In other words, for steady visual motion discrimination choice confidence was constant, as well. Possibly this finding indicates that the cortical representation of learning effects in perceptual sensitivity and metacognitive confidence in it are to some extent similar.

Since accuracy and confidence in it evolved concordantly, we next examined how precise the subjects managed to align objective performance and metacognitive reports. Did the metacognitive evaluation of the subjects’ own perceptual performance get more “valid” in the course of learning? Our findings rather demonstrate that the metacognitive sensitivity of each perceptual decision did not change significantly in the course of learning (Fig 3A). We here provide evidence for a parallel increase of perceptual sensitivity and confidence in it on the one hand, and a relative invariance of metacognitive sensitivity in the course of perceptual learning on the other. These experimental results supplement recent studies on visual perception using ROC statistics that found observers’ metacognitive sensitivity to be less than what is predicted by their objective sensitivity [17] and suggested that different representations of the same visual motion signal are read out for confidence estimates relative to sensitivity [58]. Notably, in a previous experiment using a paradigm similar to the one applied here we found even perceptual confidence to dissociate from sensitivity in visual motion discrimination with selective attention (with a significantly stronger influence of voluntary top-down attention on metacognitive than on objective performance) [9].

But did the objective performance, the subjective confidence in it, or the precision of the metacognitive evaluation guide perceptual learning? Visual perceptual learning has been reported to be modulated by various top-down cognitive factors such as selective attention [14] and performance feedback [15]. The impact of the high-level metacognitive process of decision confidence on perceptual learning, however, has not been examined yet. We here analyzed the performance gradients in motion discrimination between the nine learning sections in reference to the mean accuracy, confidence in it, or metacognitive sensitivity of the respective preceding section. In this exploration of the incremental perceptual learning effects we found that the preceding discrimination accuracy determines the extent of perceptual learning. The apparent impact of perceptual certainty (Fig 6B) on the learning performance, in contrast, resulted from its covariance with discrimination accuracy. Metacognitive sensitivity did not determine perceptual learning either (Fig 6C). In a variation of this analysis we related the learning effect over the entire experiment to initial discrimination, confidence, and metacognitive precision. The observed effects resembled those of the analysis of the incremental learning progress. They persisted when the analysis was limited to the overall learning progress from section two to nine, in order to preclude procedural learning at the beginning of the experiment from confounding the perceptual learning success of the naive subjects.

Together, according to our findings the sensitivity of a metacognitive evaluation based on perceptual choice certainty–or decision confidence itself − does not guide visual perceptual learning when analyzed on a fine scale. In line with previous data on human metacognition in perceptual learning [3], our results do not further support the notion that sensory plasticity is controlled by high-level cognitive processes. To what extent subjects learn to discriminate coherent from incoherent visual motion is determined primarily by the objective perceptual sensitivity at the outset of our learning experiment. Complementing previous findings on visual perceptual learning [55], visual motion discrimination apparently improves without the subject being consciously aware whether her perceptual decisions are right or wrong. Potentially the high number of experimental repetitions alone suggests ecological relevance and increases perceptual efficiency, similar to visual statistical learning without awareness [59] or subliminal visual priming [60].

Our findings show that sensitivity and metacognitive confidence are not fixed, but perceptual thresholds decrease with training. Neither decision confidence nor bias-free metacognitive sensitivity is requisite for changes in visual perceptual sensitivity. Choice certainty estimates rather covary with objective performance.

On a cautionary note, however, these findings apply to supervised learning in our experiment including a positive reinforcement signal by the wager feedback. Possibly these reinforcement signals enhance bottom-up visual signals and thereby interfere with top-down metacognitive influences on the learning progress. Chances are that choice certainty is critically important when we make decisions without immediate external feedback. In addition, perceptual learning effects vary considerably depending on feedback conditions, with different performance improvements for correct, manipulated or no feedback at all [61]. Giving accurate feedback here is not always the most effective way to maximize human learning [62]. That is why a strategy of selective feedback–even to the point of uncoupling feedback from the factual performance–is particularly promising. We propose future studies targeting perceptual learning while feedback conditions are systematically manipulated. The opportunity to enhance perceptual and sensorimotor learning by targeting metacognitive evaluations of a patient’s own performance could pave the way for new rehabilitative strategies.
